# Effects of neoadjuvant radiochemotherapy for anorectal function in locally advanced rectal cancer patients: a study protocol for a prospective, observational, controlled, multicentre study

**DOI:** 10.1186/s12885-023-10951-x

**Published:** 2023-05-22

**Authors:** Jie Shi, Yi-Kan Cheng, Fang He, Jian Zheng, Yun-Long Wang, Xiang-Bo Wan, Hong-Cheng Lin, Xin-Juan Fan

**Affiliations:** 1 Department of Radiation Oncology, The Sixth Affiliated Hospital, Sun Yat-sen University, Guangzhou, Guangdong 510655 P.R. China; 2 Department of General Surgery, Guangdong Provincial Key Laboratory of Colorectal and Pelvic Floor Diseases, The Sixth Affiliated Hospital, Sun Yat-sen University, Guangzhou, Guangdong 510655 P.R. China; 3 Department of Coloproctology, The Sixth Affiliated Hospital, Sun Yat-sen University, Guangzhou, Guangdong 510655 P.R. China; 4 Department of Pathology, The Sixth Affiliated Hospital, Sun Yat-sen University, Guangzhou, Guangdong 510655 P.R. China

**Keywords:** Anorectal function, Locally advanced rectal cancer, Neoadjuvant chemoradiotherapy, Total mesorectal excision, Sphincter preservation treatments

## Abstract

**Background:**

Neoadjuvant chemoradiotherapy (NCRT) and total mesorectal excision are standard treatment regimen for patients with locally advanced rectal cancer (LARC). This sphincter-saving treatment strategy may be accompanied by a series of anorectal functional disorders. Yet, prospective studies that dynamically evaluating the respective roles of radiotherapy, chemotherapy and surgery on anorectal function are lacking.

**Patients/design:**

The study is a prospective, observational, controlled, multicentre study. After screening for eligibility and obtaining informed consent, a total of 402 LARC patients undergoing NCRT followed by surgery, or neoadjuvant chemotherapy followed by surgery, or surgery only would be included in the trial. The primary outcome measure is the average resting pressure of anal sphincter. The secondary outcome measures are maximum anal sphincter contraction pressure, Wexner continence score and low anterior resection syndrome (LARS) score. Evaluations will be carried out at the following stages: baseline (T1), after radiotherapy or chemotherapy (before surgery, T2), after surgery (before closing the temporary stoma, T3), and at follow-up visits (every 3 to 6 months, T4, T5……). Follow-up for each patient will be at least 2 years.

**Discussion:**

We expect the program to provide more information of neoadjuvant radiotherapy and/or chemotherapy on anorectal function, and to optimize the treatment strategy to reduce anorectal dysfunction for LARC patients.

**Trial registration:**

ClinicalTrials.gov (NCT05671809). Registered on 26 December 2022.

## Introduction

### Background and rationale

Rectal cancer represents an important public health problem that compromises patients’ health-related quality of life. Worldwide in 2020, the incidence of rectal cancer was 3.8%, while its mortality rate was 3.4% [1]. Currently, total mesorectal excision (TME) is the gold standard method in sphincter-preserving therapy for locally advanced rectal cancer (LARC) [2, 3]. Neoadjuvant chemoradiotherapy (NCRT) is being used in patients with LARC (T3–T4 or N0–N1), with the purpose of reducing the local recurrence rates and increasing the likelihood of sphincter preservation [4, 5]. However, NCRT, especially neoadjuvant radiotherapy, and sphincter-saving operations are always criticized to cause a series of functional disorders, including high bowel frequency, urgency, and fecal incontinence (FI) [6–9]. These functional disorders are associated with worsening patients’ quality of life (QOL) [10, 11].

Indeed, both radiotherapy and surgery may greatly disturb anorectal function [12, 13]. However, how and to which extent the NCRT and TME on the anorectal dysfunction is still unclear. Anorectal manometry is the preferred technique to provide objective evaluation about the function of anorectal since it can identify functional sphincter weakness, poor rectal compliance, and rectal sensation impairment [14, 15]. In addition, the Wexner continence score is usually used to subjectively determine the degree of FI [16, 17]. Low anterior resection syndrome (LARS) score is another tool for the subjective evaluation of anorectal function [18, 19]. Previous studies have evaluated short-term or long-term effects of NCRT and/or surgery on the anorectal function of patients with rectal cancer through anorectal manometry, Wexner continence score and LARS score [20–22]. Particularly, prospective studies dynamically evaluating the different roles of radiotherapy, chemotherapy and surgery on anorectal function are lacking. Dynamic evaluation of the anorectal function might have implications for alleviating functional disorders and improving QOL by intervening treatments for sphincter-saving patients.

### Objectives

This prospective, observational, controlled, multicentre study aims to dynamically evaluate the different roles of NCRT with surgery, neoadjuvant chemotherapy (NCT) with surgery and surgery only on the anorectal function of patients with LARC by using anorectal manometry, Wexner continence score and LARS score. In addition, the correlation between manometric findings and Wexner continence score or LARS score after different treatment regimens would be investigated.

### Methods

#### Study design and setting

**Fig. 1:**
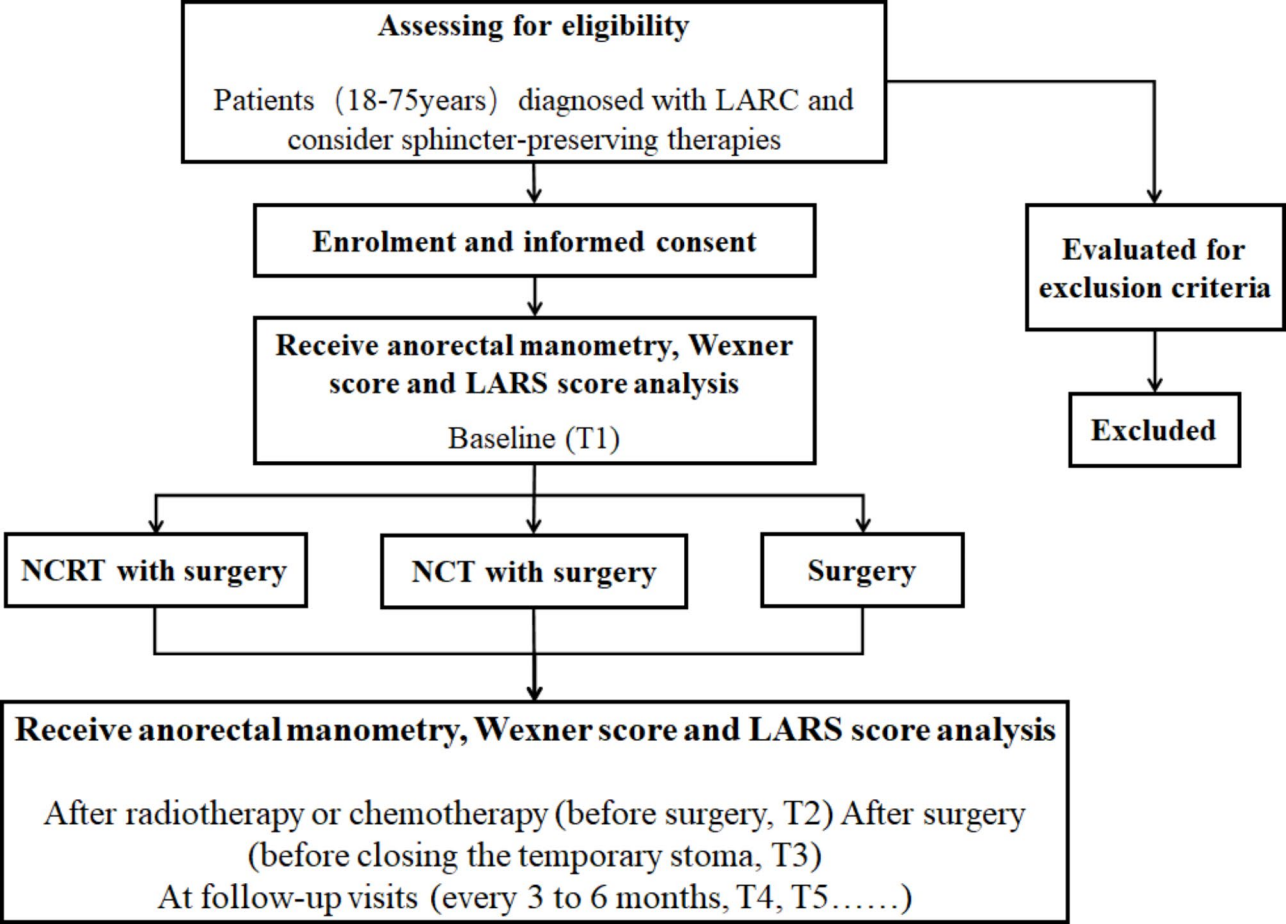
Flow diagram of the study design

The study is a prospective, observational, controlled, multicentre study of patients diagnosed with LARC. The eligible patients will be informed about the study in detail. After providing written informed consent, these patients will undergo sphincter-preserving therapies including either NCRT with surgery, or NCT with surgery or surgery only. All patients will receive anorectal manometry, Wexner continence score and LARS score before and after the treatment and at follow-up visits. The acquired data will be finally analyzed (Fig. 1).

#### Participants

Patients diagnosed with LARC at The Sixth Affiliated Hospital, Sun Yat-sen University and Nanfang Hospital, Southern Medical University will be assessed for suitability for inclusion.

#### Inclusion criteria

- 18–75 years of age.
- LARC with pathological diagnosis.
- Patient will undergo sphincter-preserving therapies.
- Eastern Cooperative Oncology Group (ECOG) score for performance status is 0–2.
- Written informed consent.

#### Exclusion criteria

- Patients who have undergone pelvic surgery, such as rectal cancer surgery and gynecological procedures.
- Patients who have received pelvic radiotherapy.
- Patients with other active malignant tumors.
- Patients with a history of prior peri-anal abscess, anorectal trauma, and inflammatory bowel disease (IBD).
- Recently (less than 4 weeks) received surgery or patients with recent severe trauma.
- Significant cardiac disease: congestive heart failure of New York Heart Association class ≥ 2; patients with recent (less than 12 months) active coronary artery disease (unstable angina or myocardial infarction).
- Recent (less than 6 months) thrombosis or embolism events, such as cerebrovascular accident (including transient ischemic attack), pulmonary embolism and deep vein thrombosis.
- Patients with toxicity (Common Terminology Criteria for Adverse Events (CTCAE) Grade ≥ 2) caused by previous treatment that has not subsided.
- Women who suffered anal sphincter tear after vaginal delivery.
- Pregnant or lactating women.

#### Interventions

The eligible patients who voluntarily sign the consent form will undergo either NCRT with surgery, or NCT with surgery, or surgery only according to treatment guidelines. The protocol for neoadjuvant radiotherapy with conventional fractionation is as follows: 1.8-2.0 Gy per day per fraction from Monday to Friday for a total of 25 fractions and a total dose of 45–50 Gy during cycles 2–4 of neoadjuvant chemotherapy. The neoadjuvant chemotherapy scheme consisted of oxaliplatin 85 mg/m^2^ intravenously, leucovorin 400 mg/m^2^ intravenously, fluorouracil 400 mg/m^2^ intravenously, and fluorouracil 2.4 g/m^2^ for 48 h continuously intravenous infusion, to be repeated every 14 days for a total of 5–6 cycles. Surgery will be performed according to the standard total mesorectal excision principles.

#### Outcomes

Evaluations will take place at baseline (T1), after radiotherapy or chemotherapy (before surgery, T2), after surgery (before closing the temporary stoma, T3), and at follow-up visits (every 3 to 6 months, T4, T5……). Follow-up for each patient will be at least 2 years.

The primary outcome is the average resting pressure (ARP) of anal sphincter. The secondary outcomes are maximum anal sphincter contraction pressure, Wexner continence score and LARS score. We will use high-resolution anorectal manometry (Solar GI HRM; MMS, Enschede, the Netherlands) to assess the average resting pressure and maximal contraction pressure, which has proven to be adequate for clinical use [23, 24]. This test will be performed by the trained physicians. In addition, we will use Wexner continence score and LARS score to assess fecal incontinence symptoms of patients following sphincter-preserving rectal cancer surgery.

### High-resolution anorectal manometry examination procedure

Bowel preparation is routinely used before the examination. The patient is placed in the left lateral position with knees and hips bent at a 90° angle, and the lubricated probe is gently inserted into the rectum. Once positioned, the probe assembly remains stationary for the duration of the study. The procedure included assessment of average resting pressure and maximum anal sphincter contraction pressure.

### Other information

Table 1 shows the information that is collected in addition to manometric findings and Wexner continence score. The study physicians will record each patient’s baseline and main examination data.

**Table 1:** Collected information

	Baseline (T1)	After radiotherapy or chemotherapy (before surgery, T2)	After surgery (before closing the temporary stoma, T3)	Follow-up (every 3 to 6 months, T4, T5……)
Age				
Sex				
BMI				
ECOG				
Colonoscopy				
CT/PET-CT scan				
MRI scan				
TNM stage				
Defecography				
Tumor marker				

BMI, Body Mass Index; ECOG, Eastern Cooperative Oncology Group; CT, Computerized Tomography; PET, Positron Emission Tomography; MRI, Magnetic Resonance Imaging; TNM, Tumor, Node and Metastasis.

### Recruitment

Patients aged 18–75 years who have been diagnosed with LARC and consider sphincter-preserving therapies are eligible for the study. After obtaining informed consent, the patient will be recruited to the study.

### Allocation

The study is a prospective, observational, controlled study without randomization. Treatment decisions will be made by multidisciplinary team for eligible patients who voluntarily sign the consent form. All patients will receive evaluation of the anorectal function through anorectal manometry, Wexner continence score and LARS score before and after therapies and at follow-up visits.

### Questionnaire used in the study

The questionnaire we use are Wexner continence score (Table 2) and LARS score (Table 3), which will be writing in Chinese thus patients can use their native language.

**Table 2:** Wexner continence score

Type of Incontinence	Frequency				
	Never	Rarely	Some- times	Usually	Always
Solid	0	1	2	3	4
Liquid	0	1	2	3	4
Gas	0	1	2	3	4
Wears pad	0	1	2	3	4
Lifestyle alteration	0	1	2	3	4

0 = normal, 1–8 = minor incontinence, 9–14 = average incontinence, 15–20 = complete incontinence.

Never = 0 (never).

Rarely = < l/month

Sometimes = < l/week,_>l/month

Usually = < l/day, _>l/week

Always = _>l/day

**Table 3:** Low anterior resection syndrome (LARS) score

The aim of this questionnaire is to assess your bowel function. Please tick only one box for each question. It may be difficult to select only one answer, as we know that for some patients symptoms vary from day to day. We would kindly ask you to choose one answer which best describes your daily life. If you have recently had an infection affecting your bowel function, please do not take this into account and focus on answering questions to reflect your usual daily bowel function.	
**Do you ever have occasions when you cannot control your flatus (wind)?**	
□ No, never □ Yes, less than once per week □ Yes, at least once per week	0 4 7
**Do you ever have any accidental leakage of liquid stool?**	
□ No, never □ Yes, less than once per week □ Yes, at least once per week	0 3 3
**How often do you open your bowels?**	
□ More than 7 times per day (24 h) □ 4–7 times per day (24 h) □ 1–3 times per day (24 h) □ Less than once per day (24 h)	4 2 0 5
**Do you ever have to open your bowels again within one hour of the last bowel opening?**	
□ No, never □ Yes, less than once per week □ Yes, at least once per week	0 9 11
**Do you ever have such a strong urge to open your bowels that you have to rush to the toilet?**	
□ No, never □ Yes, less than once per week □ Yes, at least once per week	0 11 16
**Total Score**:	
**Interpretation: 0–20 = No LARS, 21–29 = Minor LARS, 30–42 = Major LARS**	

### Participant timeline

Recruitment started in January 2023 at The Sixth Affiliated Hospital, Sun Yat-sen University and Nanfang Hospital, Southern Medical University. The data collected from the participants and the follow-up timeline are presented in Table ​1.

### Sample size

The sample size calculation was performed considering the results of a previous case-control study with rectal cancer patients undergoing chemoradiotherapy and/or surgery [25]. In this study, RP was significantly lower in the chemoradiotherapy group than in the surgery group (32.7 +/- 17 vs. 45.3 +/- 18 mmHg; P = 0.03) at the time of ileostomy closure. These values were introduced at PASS v11 software (NCSS, LLC. Kaysville, Utah, USA) with a power of 80%, alpha 0.05, and an enrollment ratio of 1/1, resulting in an estimated sample size of 122 participants in each group. Then, considering the need of lost to follow-up, which is estimated at around 10.0% of the cases, a total of 134 patients in each group will be required in this study.

### Data management, collection and monitoring

All protocol-required information collected during the study will be entered by the investigator in the electronic case report forms (CRF). The investigator should complete the CRF as soon as possible after information is collected. An explanation should be given for all missing data. The completed CRF will be reviewed and signed by the investigator. The main investigator will continuously monitor data. Data will be stored in the secured network of Sun Yat-sen University and for security reasons, in an external hard drive which will be used to back up regularly the database.

### Statistical methods

For statistical analysis of the quantitative variables with normal distribution, the mean, standard deviation (SD), median and interquartile range will be calculated. Group comparisons will be made using t tests or Mann-Whitney U test for continuous variables. Associations between the categorical variables will be tested with the Chi-Square-test or the Fisher exact test, when appropriate. Paired values (before and after therapies) will be compared for each patient using a paired t test or a Wilcoxon test. The data will be analyzed using IBM SPSS Statistics for Windows, version 27.0 (IBM Corporation, Armonk, NY, USA). A significance threshold of p < 0.05 will be adopted for all tests.

### Research ethic approval

The study adheres to the Declaration of Helsinki on medical research protocols and ethics. The protocol was reviewed and approved by the Human Medical Ethics Committee of the Sixth Affiliated Hospital of Sun Yat-sen University (number 2022ZSLYEC-614).

### Confidentiality

Patient confidentiality will be strictly maintained.

### Dissemination policy

The final study results will be published in peer-reviewed scientific journals. Furthermore, results will be communicated through professional meetings and the media.

## Discussion

This paper presents a protocol for a prospective, observational, controlled, multicentre study to dynamically evaluate the different roles of NCRT with surgery, NCT with surgery and surgery only on the anorectal function of LARC patients. There are similar studies published, but their main objective was to clarify the short-term or long-term contribution of neoadjuvant chemoradiotherapy and/or surgery on the impairment of anorectal function in patients with rectal cancer [12, 25, 26]. The most recent study has reported that the rectal adenocarcinoma patients undergoing neoadjuvant therapy were submitted to functional evaluation by anorectal manometry and the degree of FI using the Wexner continence score, before and eight weeks after NCRT [27]. Besides that, a prospective study has performed anorectal manometry preoperatively and a median of 384 days postoperatively to evaluate the impact of NCRT on anal sphincter function [28].

Major anorectal dysfunction is common in patients with rectal cancer undergoing sphincter-saving treatment. Long-term follow-up of patients and continued symptom assessment is necessary to improve the treatment of major anorectal dysfunction. Unlike most previous studies, our study will dynamically assess the changes of anorectal function in patients with rectal cancer before and after sphincter-saving treatment and at follow-up visits and further evaluate the different roles of NCRT, NCT and surgery in determining anorectal dysfunctions. Focusing on the dynamic effects of different treatments for anorectal function will provide more information on therapeutical options and strategies to reduce anorectal dysfunction.

Our study will use high-resolution anorectal manometry to evaluate anorectal function, which is widely used in clinical studies. Although Dulskas A et al. found that anorectal manometry might be insufficient to properly capture LARS [29]. Kitaguchi D and colleagues reported that high-resolution anorectal manometry was reliable for the evaluation of anorectal function after intersphincteric resection [13]. Hence, anorectal manometry can be used as an appropriate tool for evaluating anorectal dysfunctions in patients with rectal cancer.

There are some limitations in this study. The study design is observational but not randomized and blinded. At the end of the study, we will stratify the patients according to age, sex, and cancer stage. After completing the recruitment and preliminary evaluation, we will further determine whether to enlarge the sample size or not.

## Trial status

The trial recruitment started on 1 January 2023, and it is estimated to be complete by the end of December 2026.

## Data Availability

The datasets generated or analyzed during the current study are not publicly available due to the laws on privacy protection but are available from the corresponding author on reasonable request.

## Abbreviations

TME: Total mesorectal excision
LARC: Locally advanced rectal cancer
NCRT: Neoadjuvant chemoradiotherapy
FI: Fecal incontinence
QOL: Quality of life
LARS: Low anterior resection syndrome
NCT: Neoadjuvant chemotherapy
ECOG: Eastern Cooperative Oncology Group
IBD: Inflammatory bowel disease
CTCAE: Common Terminology Criteria for Adverse Events
ARP: Average resting pressure
BMI: Body Mass Index
CT: Computerized Tomography
PET: Positron Emission Tomography
MRI: Magnetic Resonance Imaging
TNM: Tumor, Node and Metastasis
CRF: Case report forms
SD: Standard deviation
